# Supplementary material for: Whole-genome doubling drives oncogenic loss of chromatin segregation
Source: Nature. 2023 Mar 15;615(7954):925–33. doi: 10.1038/s41586-023-05794-2 (PMC10060163; doi:10.1038/s41586-023-05794-2)
Supplement: Supplementary file 1 — This file contains Supplementary Figs. 1–6. [file 41586_2023_5794_MOESM1_ESM.pdf]

nature portfolio

https://doi.org/10.1038/s41586-023-05794-2

Supplementary information

Whole-genome doubling drives oncogenic loss of chromatin segregation

In the format provided by the authors and unedited

SUPPLEMENTARY INFORMATION to:

Whole genome doubling drives oncogenic loss of chromatin segregation

Ruxandra A. Lambuta[1,2,*], Luca Nanni[2,3,4,*], Yuanlong Liu[2,3,4], Juan Diaz-Miyar[1,2], Arvind Iyer[2,3,4], Daniele Tavernari[2,3,4], Natalya Katanayeva[1,2], Giovanni Ciriello[2,3,4,#], Elisa Oricchio[1,2,#]

[1] Swiss Institute for Experimental Cancer Research (ISREC), School of Life Sciences, EPFL

[2] Swiss Cancer Center Leman, Lausanne, Switzerland

[3] Department of Computational Biology, University of Lausanne (UNIL), 1015 Lausanne, Switzerland.

[4] Swiss Institute of Bioinformatics (SIB) Switzerland

* These authors equally contributed to this work

# Corresponding Authors:

Correspondence and requests for materials should be addressed to:

Elisa Oricchio e-mail: elisa.oricchio@epfl.ch

Giovanni Ciriello e-mail: giovanni.ciriello@unil.ch

Contents:

Supplementary Fig. 1. Hi-C maps in control, WGD and CIN-only models.

Supplementary Fig. 2. Quality filtering and individual scHi-C maps in control and WGD RPE$^{TP53-/-}$ cells.

Supplementary Fig. 3. Hi-C maps of 20-weeks post-WGD tumours.

Supplementary Fig. 4. Ploidy evolution of CP-A$^{TP53-/-}$ post-WGD cells.

Supplementary Fig. 5: Uncropped immunoblots present in main figures.

Supplementary Fig. 6: Gating strategy for the flow cytometry-based cell cycle analyses.

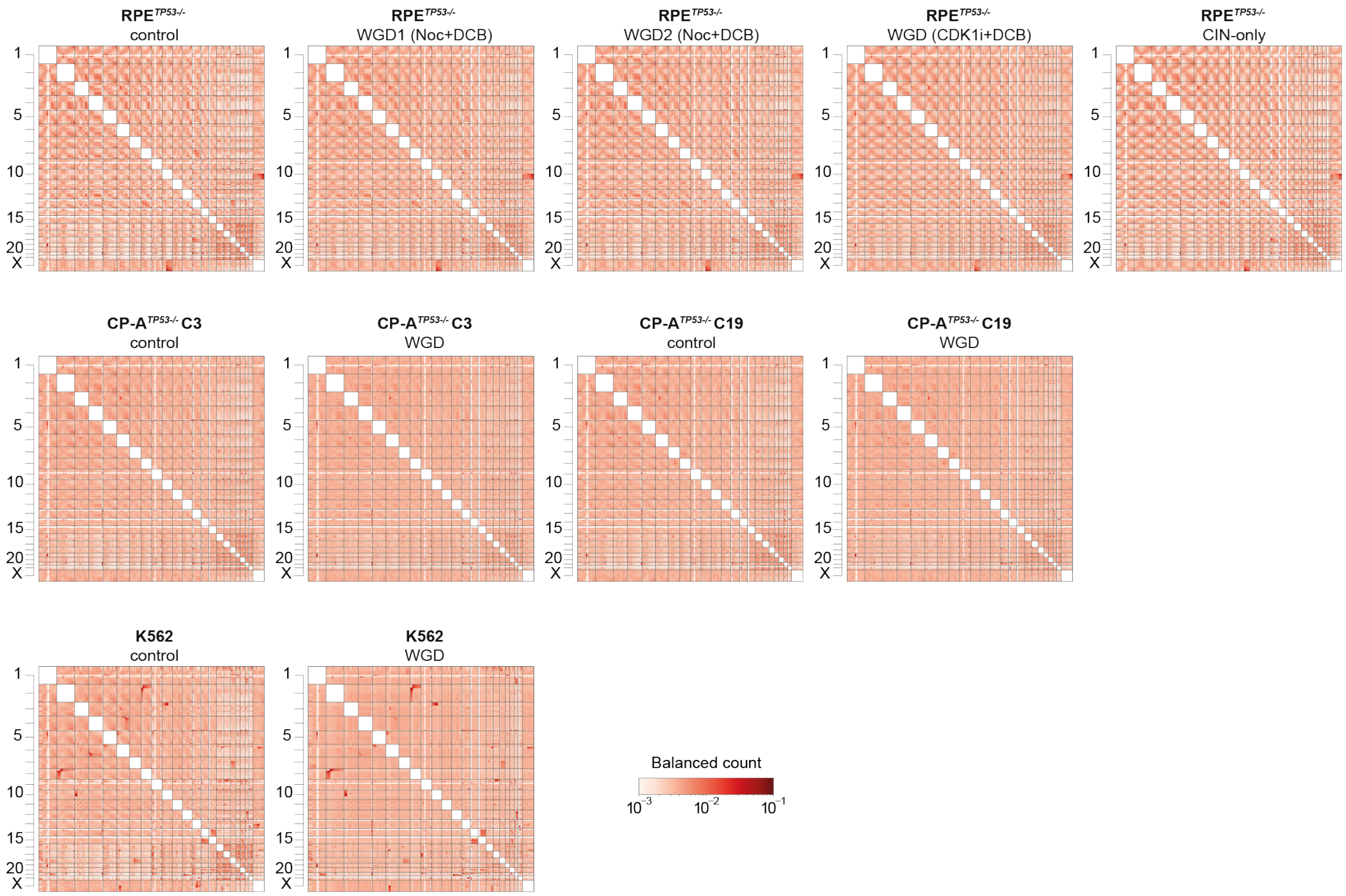

Supplementary Fig. 1: Hi-C maps in control, WGD and CIN-only models. Hi-C inter-chromosomal interaction maps at 10Mb resolution. Matrices are balanced using Iterative Correction so the inter-chromosomal marginal sum of each bin sums up to 1.

A. RPE$^{TP53-/-}$ control

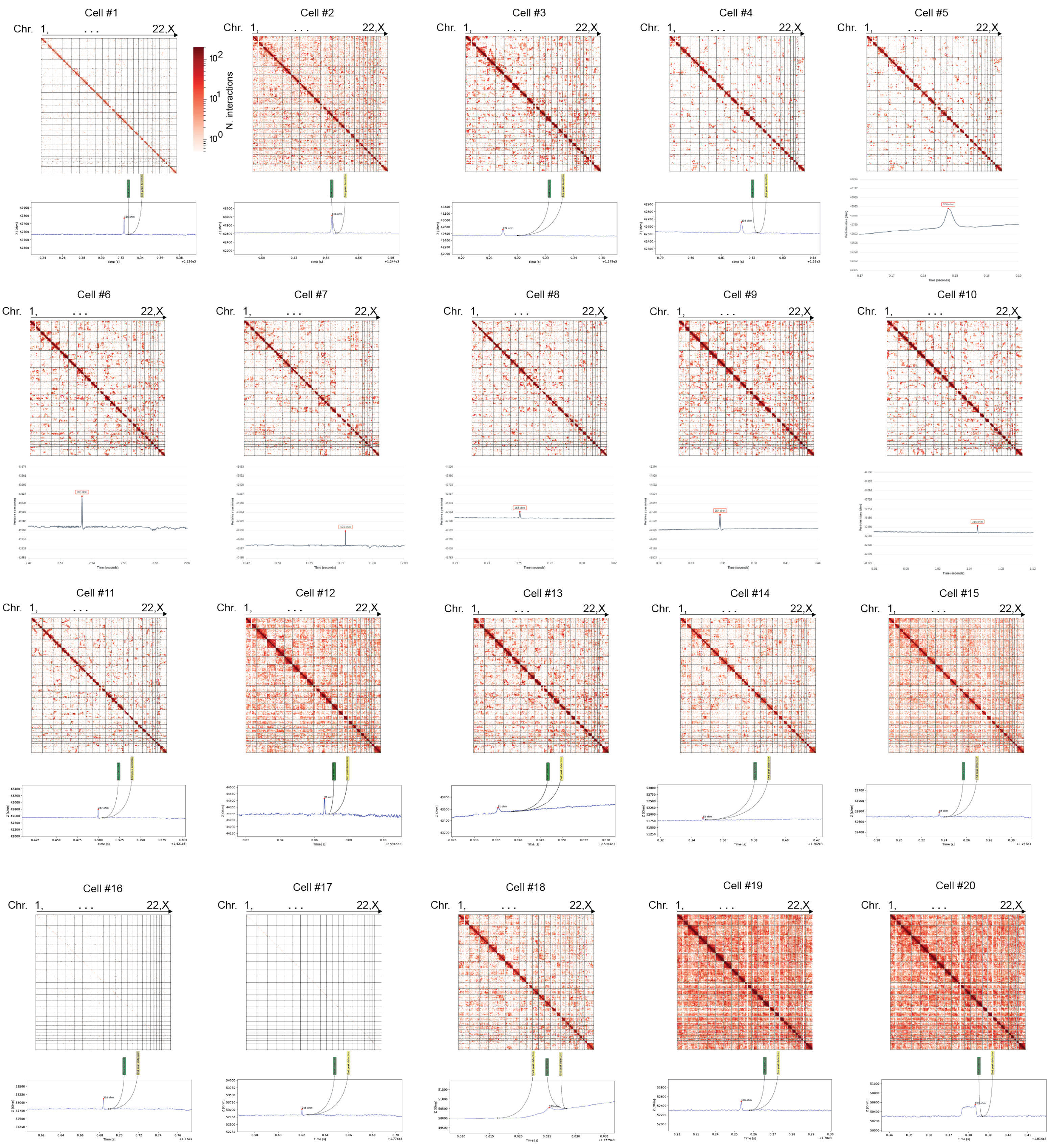

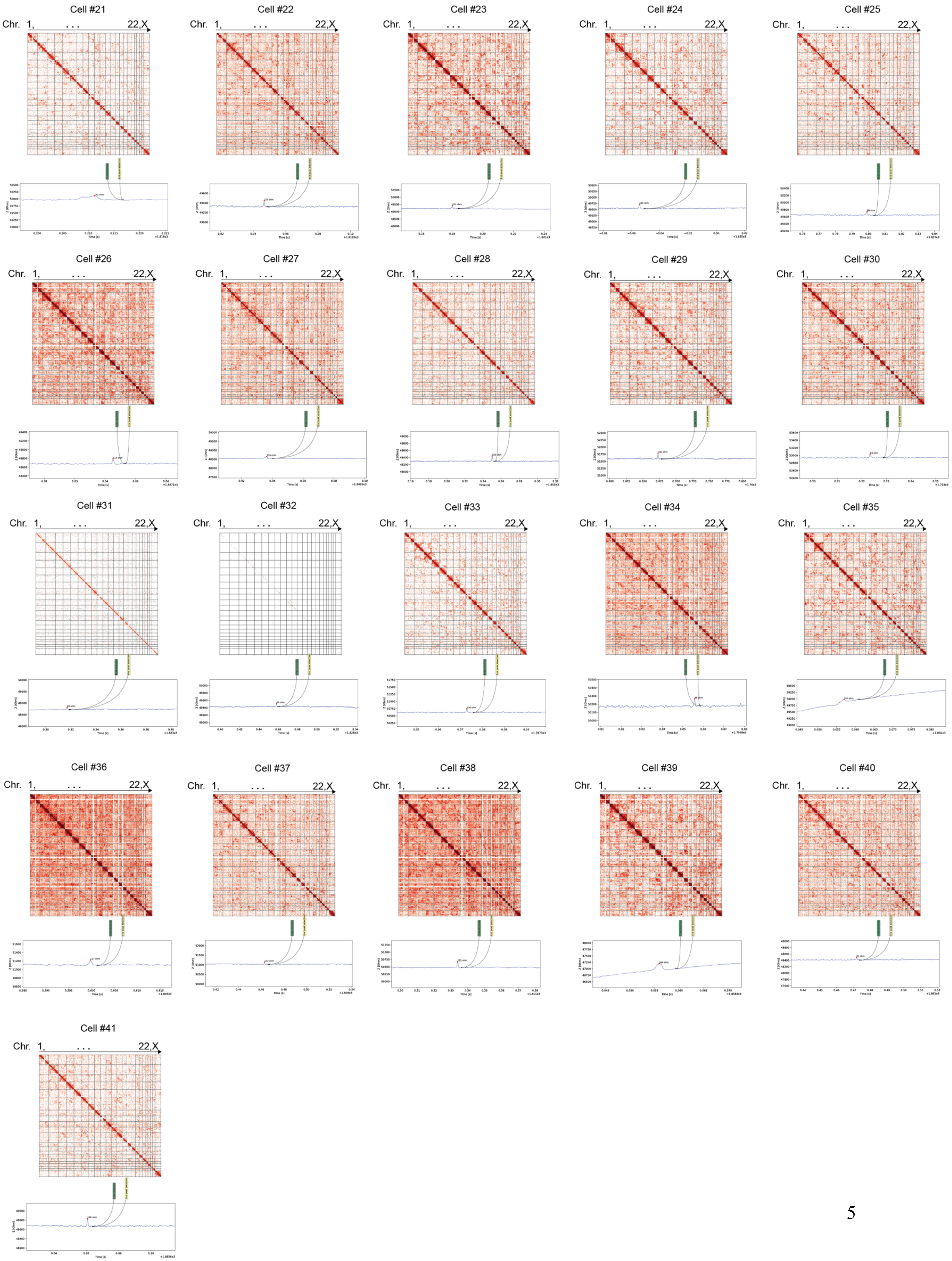
Cell #21
Cell #22
Cell #23
Cell #24
Cell #25
Cell #26
Cell #27
Cell #28
Cell #29
Cell #30
Cell #31
Cell #32
Cell #33
Cell #34
Cell #35
Cell #36
Cell #37
Cell #38
Cell #39
Cell #40
Cell #41
Chr. 1, . . . 22,X

B. RPE$^{TP53-/-}$ WGD

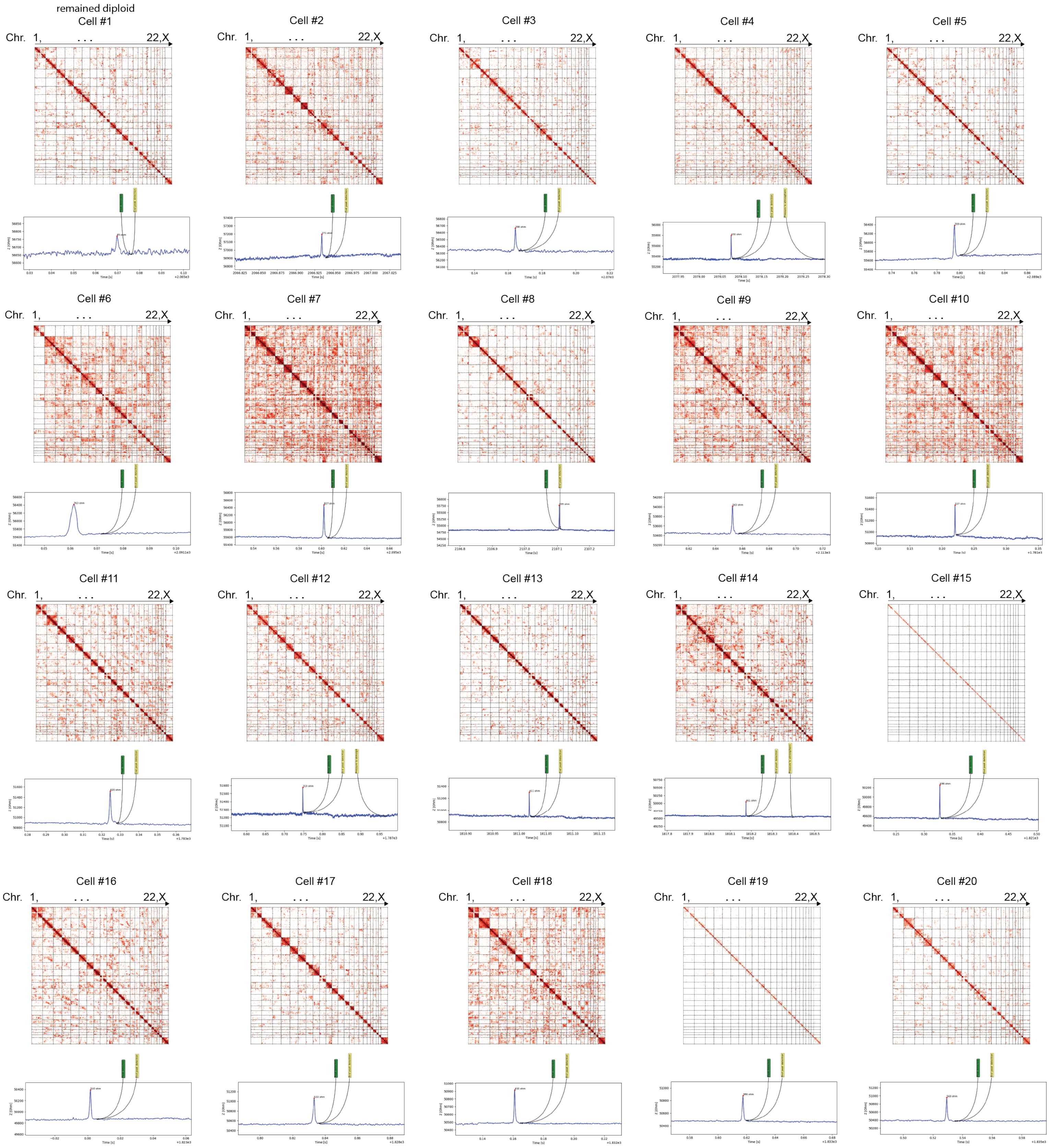

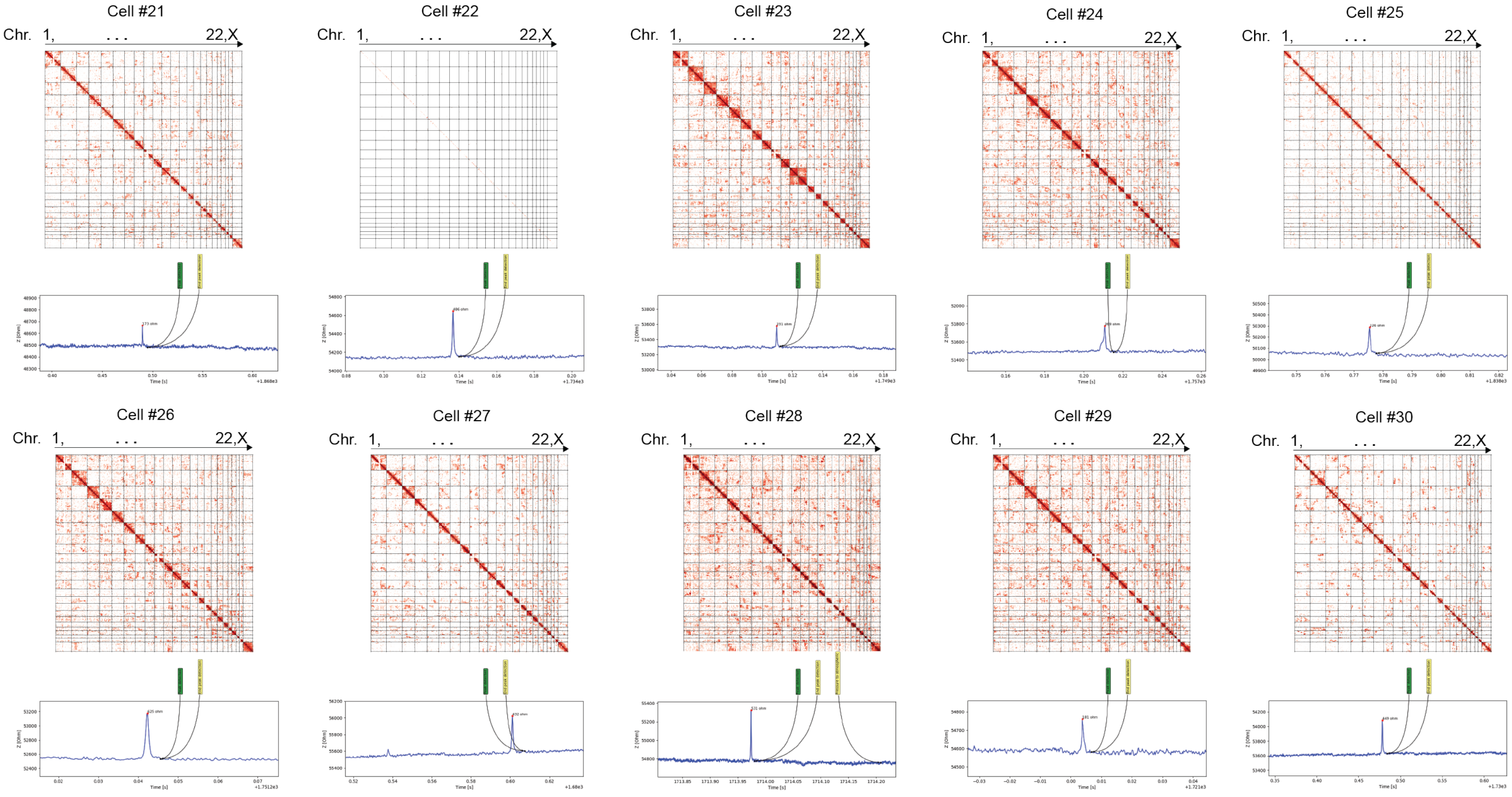

Supplementary Fig. 2: Quality filtering and individual scHi-C maps in control and WGD RPE$^{TP53-/-}$ cells. Raw Hi-C maps and impedance-based single nucleus sorting signal (ohm) for each single cell in the RPE$^{TP53-/-}$ control **(A)** and WGD **(B)** collections.

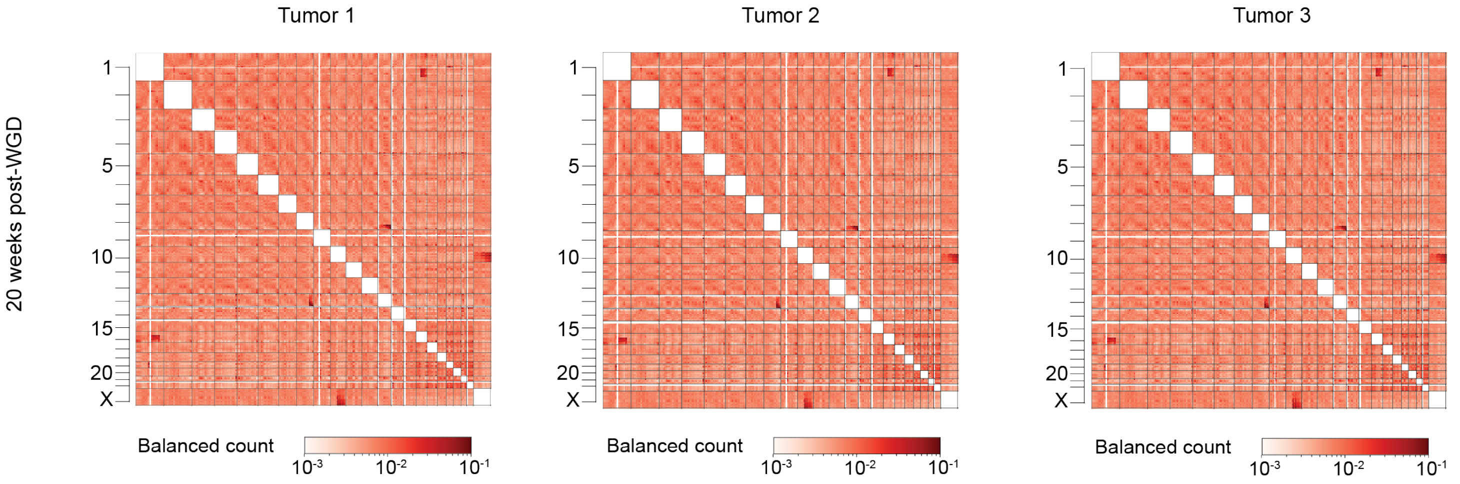

Supplementary Fig. 3: Hi-C maps of 20-weeks post-WGD tumours. Hi-C inter-chromosomal interaction maps at 10Mb resolution of the three 20-weeks post-WGD tumours originated from RPE$^{TP53-/-}$ cells. Matrices are balanced using Iterative Correction so the inter-chromosomal marginal sum of each bin sums up to 1.

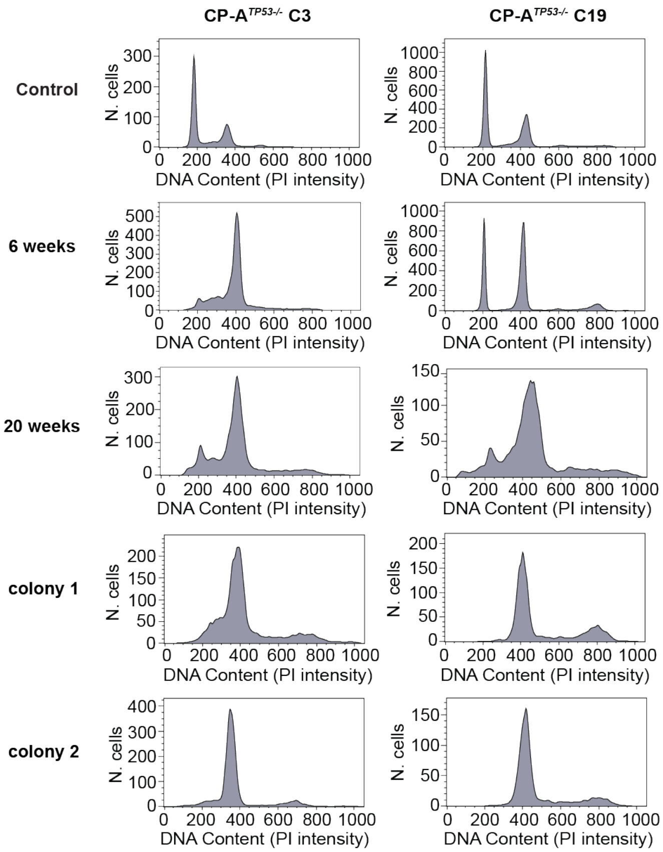

Supplementary Fig. 4: Ploidy evolution of CP-A$^{TP53-/-}$ post-WGD cells. PI-based cell cycle staining histograms of CP-A$^{TP53-/-}$ (clone 19 and clone 3) control, 6 weeks and 20 weeks post-WGD cells *in vitro*, and soft agar colonies.

Figure 2a

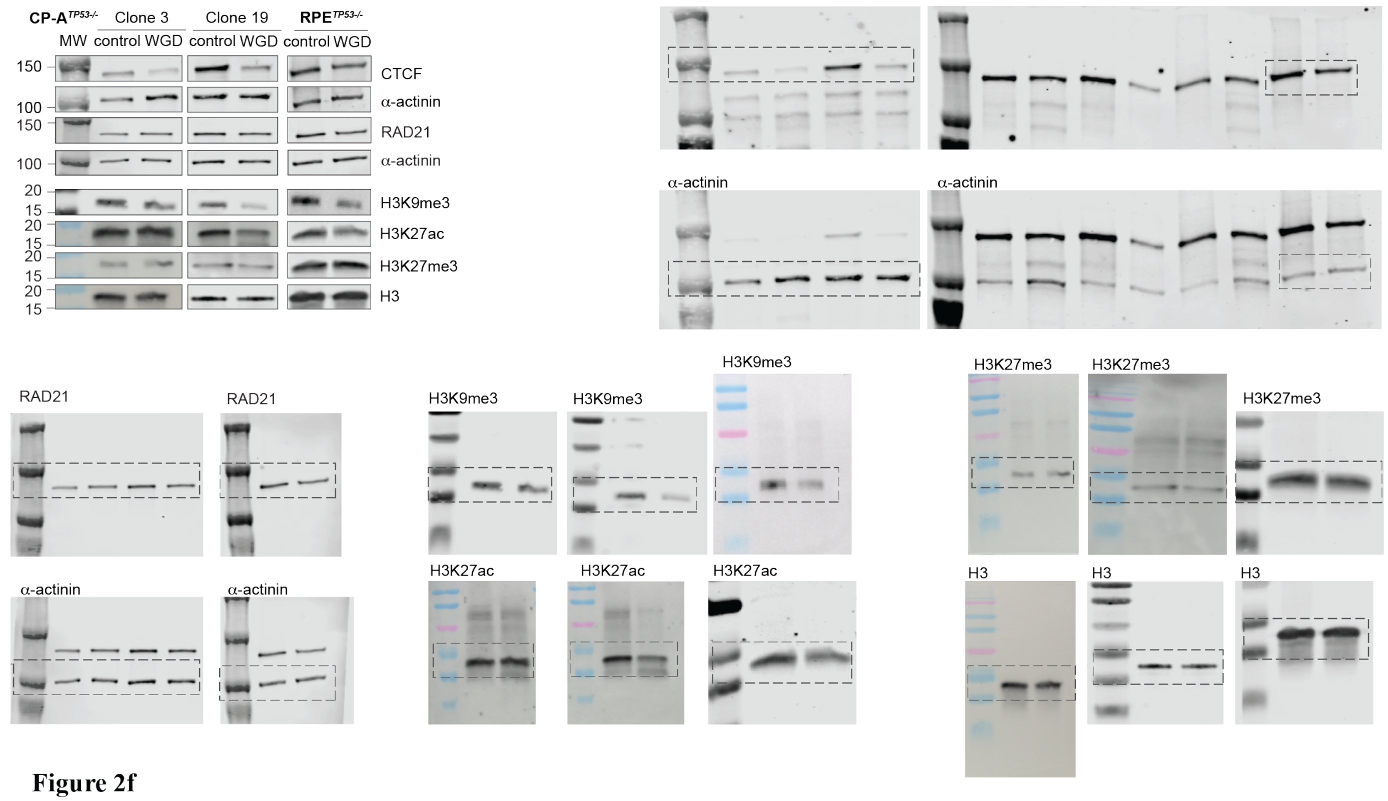

Figure 2f

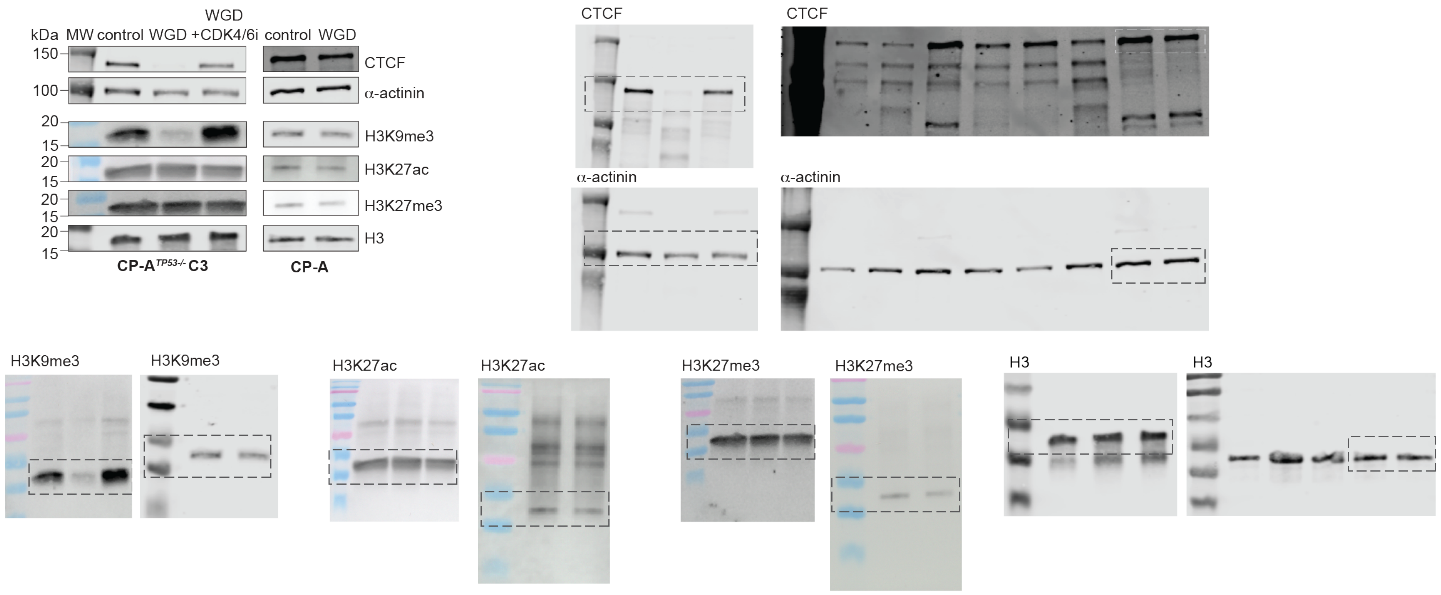

Extended Data Figure 1a

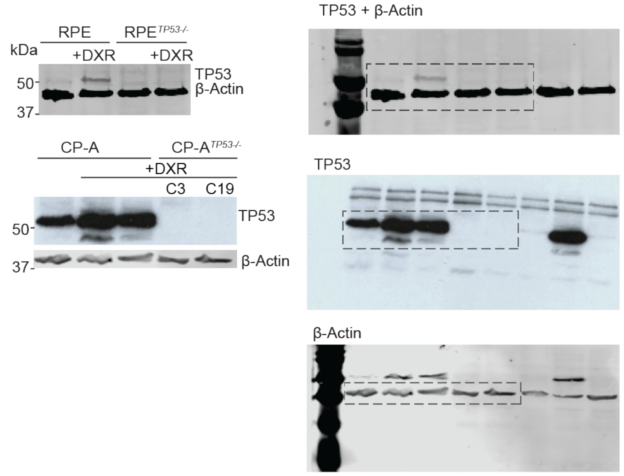

Extended Data Figure 5d

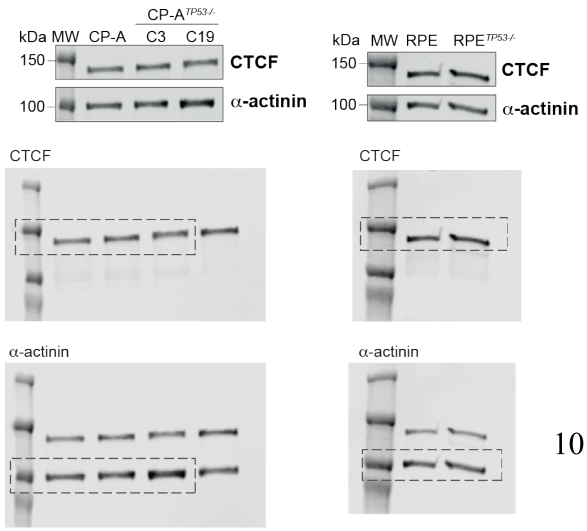

Supplementary Fig. 5: Uncropped immunoblots present in main figures.

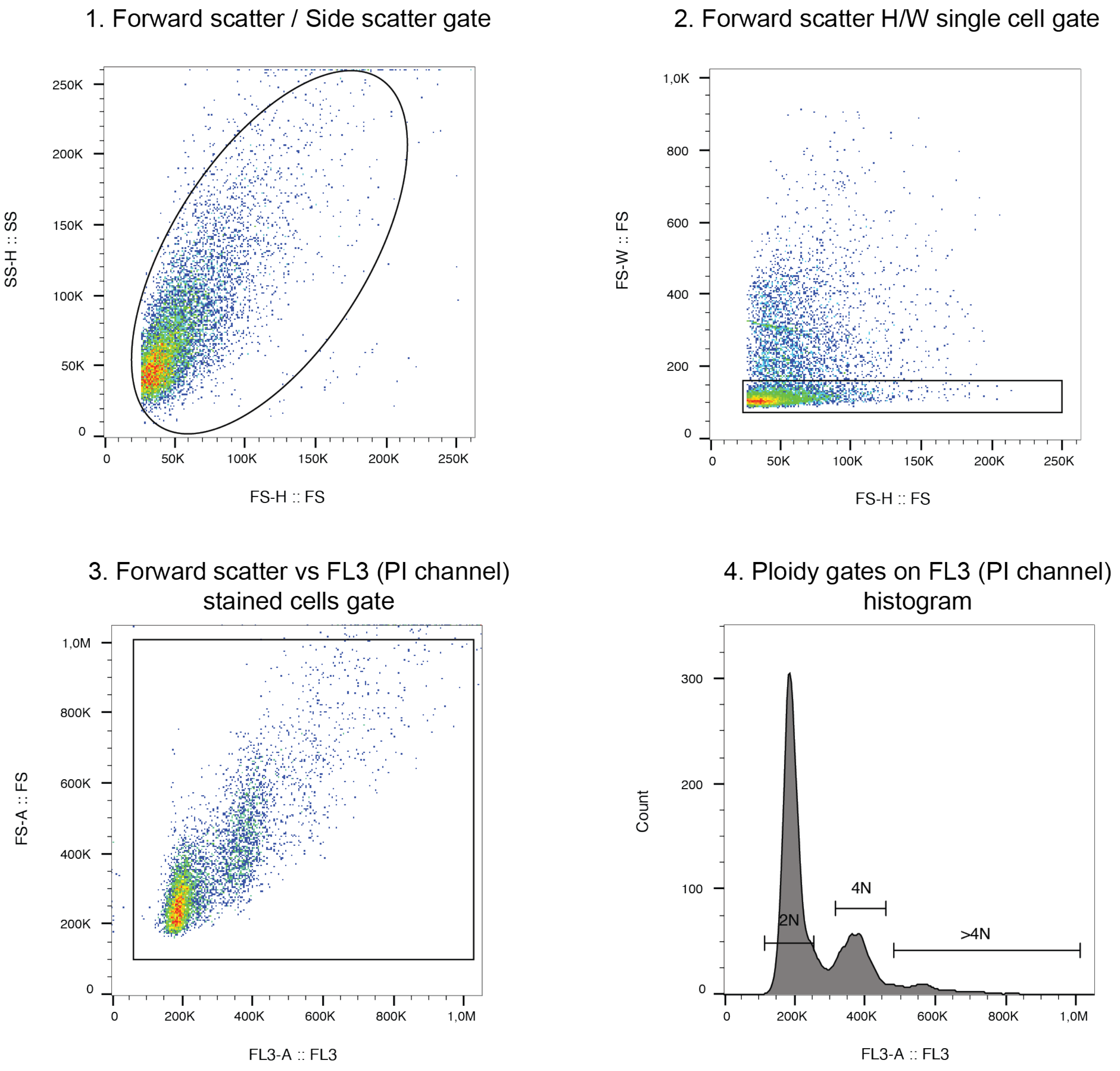

Supplementary Fig. 6: Gating strategy for the flow cytometry-based cell cycle analyses.
