# Supplementary material for: Whole-genome doubling drives oncogenic loss of chromatin segregation
Source: Nature. 2023 Mar 15;615(7954):925–33. doi: 10.1038/s41586-023-05794-2 (PMC10060163; doi:10.1038/s41586-023-05794-2)
Supplement: Supplementary file 2 — Reporting Summary [file 41586_2023_5794_MOESM2_ESM.pdf]

nature portfolio

Corresponding author(s): Elisa Oricchio
Giovanni Ciriello

Last updated by author(s): Jan 9, 2023

Reporting Summary

Nature Portfolio wishes to improve the reproducibility of the work that we publish. This form provides structure for consistency and transparency in reporting. For further information on Nature Portfolio policies, see our Editorial Policies and the Editorial Policy Checklist.

Statistics

For all statistical analyses, confirm that the following items are present in the figure legend, table legend, main text, or Methods section.

n/a	Confirmed	
☐	☒	The exact sample size (*n*) for each experimental group/condition, given as a discrete number and unit of measurement
☐	☒	A statement on whether measurements were taken from distinct samples or whether the same sample was measured repeatedly
☐	☒	The statistical test(s) used AND whether they are one- or two-sided<br>*Only common tests should be described solely by name; describe more complex techniques in the Methods section.*
☒	☐	A description of all covariates tested
☐	☒	A description of any assumptions or corrections, such as tests of normality and adjustment for multiple comparisons
☐	☒	A full description of the statistical parameters including central tendency (e.g. means) or other basic estimates (e.g. regression coefficient) AND variation (e.g. standard deviation) or associated estimates of uncertainty (e.g. confidence intervals)
☐	☒	For null hypothesis testing, the test statistic (e.g. *F*, *t*, *r*) with confidence intervals, effect sizes, degrees of freedom and *P* value noted<br>*Give P values as exact values whenever suitable.*
☒	☐	For Bayesian analysis, information on the choice of priors and Markov chain Monte Carlo settings
☒	☐	For hierarchical and complex designs, identification of the appropriate level for tests and full reporting of outcomes
☐	☒	Estimates of effect sizes (e.g. Cohen's *d*, Pearson's *r*), indicating how they were calculated

Our web collection on statistics for biologists contains articles on many of the points above.

Software and code

Policy information about availability of computer code

Data collection

Next generation sequencing was performed on Illumina Hi-SeqX, Illumina NextSeq 500, Illumina NovaSeq 6000, or Illumina HiSeq 4000.
Single cell RNA sequencing was performed on 10X Genomics Chromium.
Flow cytometry was performed on Guava easyCyte™ (Luminex) and Galios (BeckmanCoulter) cytometers.
Microscopy imaging was performed on a Zeiss Axioplan upright microscope.
Western blots were imaged with the Odyssey CLx Imaging System (LICOR) or FUSION FX6 EDGE Imaging System (Witec).

Data analysis

Analysis code to reproduce the results presented in the manuscript is available at https://github.com/CSOgroup/WGD

The following software has been used for data processing and analysis:

- STAR, version 2.7.10a
- RSEM, version 1.3.1
- DESeq2, version 1.34.0
- MAST, version 1.18.0
- Mutect2, version 4.2.2.0
- gnomAD, version 2 (b37)
- FlowJo v10.8
- Fiji v2.9.0
- Python, version 3.9.0
- R, version 4.1.0
- bwa, version 0.7.17 (alignement of reads)
- samtools, version 1.10 (analysis of reads)
- Juicer 1.6 (processing of Hi-C reads)
- hicrep.py, version 0.2.6 (calculation of the SCC score)

- FANC, version 0.9.21 (Hi-C insulation score and boundaries)
- Freebayes, version v1.3.2-46-g2c1e395-dirty (identification of SNVs from Hi-C reads for phasing)
- SHAPEIT2, version v2.904.3.10.0-693.11.6.el7.x86_64 (population-based phasing)
- HapCUT2, version 1.3.3 (second round of phasing)
- scHiCExplorer, version 7 (analysis of Single-cell Hi-C reads)
- hicexplorer, version 3.5.1 (analysis of Hi-C data)
- cooler, version 0.8.11 (analysis of Hi-C data)
- cooltools, version 0.5.0 (analysis of Hi-C data)
- cell-ranger, version 3.1.1 (analysis of scRNAseq data)
- Seurat, version v3.1.5 (analysis of scRNAseq data)
- InferCNV, version v1.1.0 (analysis of scRNAseq data)
- fastcluster, version v1.2.3 (clustering)
- matplotlib, version v3.4.2 (plotting)
- seaborn, version 0.11.2 (plotting)
- pyscenic, version 0.11.2 (analysis of scRNAseq data)
- GATK, version v4.2.2.0 (marking of duplicates, reads analysis)
- Control-FREEC, version 11.6 (CNV calling)
- bedtools, version v2.30.0 (bed file management)
- vcf2maf, version 1.6.21 (conversion from vcf to maf)
- nfcore/chipseq v.1.2.2 (ChIP-seq reads processing)
- deeptools, version 3.5.1 (analysis of ChIP-seq data)
- MACS3, version 3.0.0a6 (calling peaks from ChIP-seq data)
- nfcore/rnaseq v.3.8 (RNA-seq reads processing)
- HiC-DC, downloaded from https://bitbucket.org/leslielab/hic-dc/src/master/

For manuscripts utilizing custom algorithms or software that are central to the research but not yet described in published literature, software must be made available to editors and reviewers. We strongly encourage code deposition in a community repository (e.g. GitHub). See the Nature Portfolio guidelines for submitting code & software for further information.

Data

Policy information about availability of data

All manuscripts must include a data availability statement. This statement should provide the following information, where applicable:

- Accession codes, unique identifiers, or web links for publicly available datasets
- A description of any restrictions on data availability
- For clinical datasets or third party data, please ensure that the statement adheres to our policy

Reference genome hg19 was downloaded from http://hgdownload.cse.ucsc.edu/goldenpath/hg19/chromosomes/.
Reference genome human_g1k_hs37d5 was downloaded from http://ftp.1000genomes.ebi.ac.uk/vol1/ftp/technical/reference/phase2_reference_assembly_sequence/
The gnomAD database was downloaded from https://storage.googleapis.com/gatk-best-practices/somatic-b37/af-only-gnomad.raw.sites.vcf.
The dbSNP database was dowloaded from https://ftp.ncbi.nlm.nih.gov/snp/organisms/human_9606/VCF/00-common_all.vcf.gz
Cellranger reference data were downloaded and processed as reported at https://support.10xgenomics.com/single-cell-gene-expression/software/release-notes/build#hg19_3.0.0.
Hi-C data of the GM12878 cell line were downloaded from GSE63525

Processed Hi-C data together with compartment domain calls by Calder are available at https://doi.org/10.5281/zenodo.7351767.
ChIP-seq data, single-cell RNA-seq matrices and copy number profiles of RPE TP53-/- samples are available at https://doi.org/10.5281/zenodo.7351776.
Raw files are deposited at: GSE222390

Data available:
Hi-C (includes Hi-C maps and following features: compartment domains, insulation boundaries, insulation scores):
CP-A control
CP-A WGD
CP-A TP53-/- clone 19 control
CP-A TP53-/- clone 19 WGD
CP-A TP53-/- clone 19 post-WGD colony 1
CP-A TP53-/- clone 19 post-WGD colony 2
CP-A TP53-/- clone 3 control
CP-A TP53-/- clone 3 WGD
CP-A TP53-/- clone 3 WGD + CDK4/6i
CP-A TP53-/- clone 3 post-WGD colony 1
CP-A TP53-/- clone 3 post-WGD colony 2
CP-A TP53-/- clone 3 spontaneous high-ploidy
RPE
RPE TP53-/- control
RPE TP53-/- WGD R1
RPE TP53-/- WGD R2
RPE TP53-/- WGD (CDK1i+DCB)
RPE TP53-/- CIN-only
RPE TP53-/- 20wk Tumor 1
RPE TP53-/- 20wk Tumor 2
RPE TP53-/- 20wk Tumor 3
K562 Control
K562 WGD

ChIP-Seq (for each of the following targets: CTCF, H3K27ac, H3K27me3, H3K9me3, H3K4me3):
RPE TP53-/- Control
RPE TP53-/- 20wk Tumor 1
RPE TP53-/- 20wk Tumor 2
RPE TP53-/- 20wk Tumor 3

ChIP-Seq (for CTCF and H3K9me3):
RPE TP53-/- WGD
CP-A TP53-/- clone 3 Control
CP-A TP53-/- clone 3 WGD

WGS (CNVs):
merged file for RPE TP53-/- control, WGD, 20wk Tumor 1, 20wk Tumor 2, 20wk Tumor 3

WGS (mutations):
merged files (all mutations and filtered mutations) for RPE TP53-/- control, WGD, 6wk in vitro, 20wk in vitro, 20wk Tumor 1, 20wk Tumor 2, 20wk Tumor 3

scRNASeq (differential expression files, gene expression matrix (cell x gene), infercnv data):
RPE TP53-/- control
RPE TP53-/- 6wk in vitro
RPE TP53-/- 20wk in vitro
RPE TP53-/- 20wk Tumor 1
RPE TP53-/- 20wk Tumor 2
RPE TP53-/- 20wk Tumor 3

RNA-Seq:
RPE TP53-/- control (3 replicates)
RPE TP53-/- WGD (3 replicates)

Field-specific reporting

Please select the one below that is the best fit for your research. If you are not sure, read the appropriate sections before making your selection.

☒ Life sciences ☐ Behavioural & social sciences ☐ Ecological, evolutionary & environmental sciences

For a reference copy of the document with all sections, see nature.com/documents/nr-reporting-summary-flat.pdf

Life sciences study design

All studies must disclose on these points even when the disclosure is negative.

Sample size	No power analysis was performed to determine the correct sample size. However, three cell lines of distinct origins and distinct induction methods were used, which is sufficient for validating WGD effects on chromatin. Six to nine animals per group were used for in vivo experiments, which is sufficient for proof of tumorigenesis and downstream analyses, while also respecting 3R principles for animal research. For all other experiments, sample size was chosen in order to provide sufficient statistical power.
Data exclusions	No data or sequence were excluded, except for the low pass quality reads.
Replication	The number of independent replicate for each experiment is reported in the figure legends and/or in the text.
Randomization	No randomization was applied as it was not necessary in this study.
Blinding	For sequencing experiments, as well as for immunoblots, comparisons were performed between controls and WGD or pWGD conditions, thus blinding was not possible. For karyotyping and immunofluorescence experiments, control and WGD cells have clear distinctions (increased number of chromosomes or nucleus size), which also impedes blinding.

Reporting for specific materials, systems and methods

We require information from authors about some types of materials, experimental systems and methods used in many studies. Here, indicate whether each material, system or method listed is relevant to your study. If you are not sure if a list item applies to your research, read the appropriate section before selecting a response.

Materials & experimental systems

n/a	Involved in the study
☐	☒ Antibodies
☐	☒ Eukaryotic cell lines
☒	☐ Palaeontology and archaeology
☐	☒ Animals and other organisms
☒	☐ Human research participants
☒	☐ Clinical data
☒	☐ Dual use research of concern

Methods

n/a	Involved in the study
☐	☒ ChIP-seq
☐	☒ Flow cytometry
☒	☐ MRI-based neuroimaging

Antibodies

Antibodies used

For immunofluorescence:
anti-Pericentrin (Abcam, catalogue no. ab4448), anti-α-Tubulin (Sigma-Aldrich, catalogue no. T6074), anti-Mouse IgG-Alexa Fluor 594 (Thermo Fisher Scientific, catalogue no. A-11005), anti-Rabbit IgG-Alexa Fluor 488 (Thermo Fisher Scientific, catalogue no. A-11034).
For immunoblotting:
anti-TP53 (Santa Cruz Biotechnology, catalogue no. sc-126), anti-β-Actin (Cell Signaling Technology, catalogue no. 4967), anti-CTCF (Active Motif, catalogue no. 61311), anti-RAD21 (Abcam, catalogue no. ab992), anti-α-Actinin (Cell Signaling Technology, catalogue no. 6487), anti-Tri-Methyl-Histone H3 (Lys9) (Cell Signaling Technology, catalogue no. 13969), anti-Acetyl-Histone H3 (Lys27) (Cell Signaling Technology, catalogue no. 8173), anti-Tri-Methyl-Histone H3 (Lys27) (Cell Signaling Technology, catalogue no. 9733), anti-Histone H3 (Cell Signaling Technology, catalogue no. 4499), Goat anti-Mouse (LI-COR Biosciences, catalogue no. 926-68070), Goat anti-Rabbit (LI-COR Biosciences, catalogue no. 926-32211), HRP-conjugated Goat Anti-Mouse Antibody (Merck, catalogue no. AP308P), Goat Anti-Rabbit Antibody (Merck, catalogue no. AP307P).
For ChIP-Seq:
anti-Acetyl-Histone H3 (Lys27) (Cell Signaling Technology, catalogue no. 8173), anti-Tri-Methyl-Histone H3 (Lys9) (Cell Signaling Technology, catalogue no. 13969), anti-CTCF (Active Motif, catalogue no. 61311), anti-Tri-Methyl-Histone H3 (Lys4) (Cell Signaling Technology, catalogue no. 9751), Tri-Methyl-Histone H3 (Lys27) (Cell Signaling Technology, catalogue no. 9733).

Validation

All antibodies used in this study are commercially available and validated for the application they were used for in this study, as stated on the manufacturer's websites. Additionally, the antibodies were previously validated and used in numerous studies.

Eukaryotic cell lines

Policy information about cell lines

Cell line source(s)

hTERT RPE-1 (originally from ATCC, from the laboratory of Dr. Jan Korbel, EMBL Heidelberg, Germany)
hTERT RPE-1 TP53-/- (from the laboratory of Dr. Jan Korbel, EMBL Heidelberg, Germany)
CP-A (KR-42421) (ATCC)
CP-A TP53-/- (this study)
K562 (DSMZ)

Authentication

None of the cell lines were authenticated.

Mycoplasma contamination

All cell lines tested negative for mycoplasma contamination.

Commonly misidentified lines
(See ICLAC register)

No commonly misidentified cell lines were used.

Animals and other organisms

Policy information about studies involving animals; ARRIVE guidelines recommended for reporting animal research

Laboratory animals

All animals used in the study were NOD scid gamma (NSG) female mice maintained at the EPFL animal facilities in a 12h-light 12h-dark cycle, at 18-23°C with 40-60% humidity, as recommended and in accordance with the regulations of the Animal Welfare Act (SR 455) and Animal Welfare Ordinance (SR 455.1).
Experiment timelines are described in the manuscript.

Wild animals

This study did not involve wild animals.

Field-collected samples

The study did not involved samples collected from the field.

Ethics oversight

Animal experiments were performed in accordance with the Swiss Federal Veterinary Office guidelines and as authorized by the Cantonal Veterinary Office (animal license VD2932.1).

Note that full information on the approval of the study protocol must also be provided in the manuscript.

ChIP-seq

Data deposition

☒ Confirm that both raw and final processed data have been deposited in a public database such as GEO.

☒ Confirm that you have deposited or provided access to graph files (e.g. BED files) for the called peaks.

Data access links<br>*May remain private before publication.*	GSE222390<br>https://doi.org/10.5281/zenodo.7351767<br>https://doi.org/10.5281/zenodo.7351776
Files in database submission	Bigwig files of ChIP-seq experiments generated in this study. Files are log2 input normalized tracks. The "bw_signals_attributes.txt" file can be used to add metadata to the tracks for visualization in IGV.<br><br>Files available:<br><br>RPE_TP53_Ctrl_H3K27ac_vs_RPE_TP53_Ctrl_input.bigWig<br>RPE_TP53_Ctrl_H3K27me3_vs_RPE_TP53_Ctrl_input.bigWig<br>RPE_TP53_Ctrl_H3K4me3_vs_RPE_TP53_Ctrl_input.bigWig<br>RPE_TP53_Ctrl_H3K9me3_vs_RPE_TP53_Ctrl_input.bigWig<br>RPE_TP53_Ctrl_CTCF_vs_RPE_TP53_Ctrl_input.bigWig<br><br>RPE_TP53_WGD_H3K9me3_vs_RPE_TP53_WGD_input.bigWig<br>RPE_TP53_WGD_CTCF_vs_RPE_TP53_WGD_input.bigWig<br><br>RPE_TP53_20w0T1_CTCF_vs_RPE_TP53_20w0T1_input.bigWig<br>RPE_TP53_20w0T1_H3K27ac_vs_RPE_TP53_20w0T1_input.bigWig<br>RPE_TP53_20w0T1_H3K27me3_vs_RPE_TP53_20w0T1_input.bigWig<br>RPE_TP53_20w0T1_H3K4me3_vs_RPE_TP53_20w0T1_input.bigWig<br>RPE_TP53_20w0T1_H3K9me3_vs_RPE_TP53_20w0T1_input.bigWig<br><br>RPE_TP53_20w0T2_CTCF_vs_RPE_TP53_20w0T2_input.bigWig<br>RPE_TP53_20w0T2_H3K27ac_vs_RPE_TP53_20w0T2_input.bigWig<br>RPE_TP53_20w0T2_H3K27me3_vs_RPE_TP53_20w0T2_input.bigWig<br>RPE_TP53_20w0T2_H3K4me3_vs_RPE_TP53_20w0T2_input.bigWig<br>RPE_TP53_20w0T2_H3K9me3_vs_RPE_TP53_20w0T2_input.bigWig<br><br>RPE_TP53_20w0T3_CTCF_vs_RPE_TP53_20w0T3_input.bigWig<br>RPE_TP53_20w0T3_H3K27ac_vs_RPE_TP53_20w0T3_input.bigWig<br>RPE_TP53_20w0T3_H3K27me3_vs_RPE_TP53_20w0T3_input.bigWig<br>RPE_TP53_20w0T3_H3K4me3_vs_RPE_TP53_20w0T3_input.bigWig<br>RPE_TP53_20w0T3_H3K9me3_vs_RPE_TP53_20w0T3_input.bigWig<br><br>CPA-TP53-Clone3_Ctrl_CTCF_vs_CPA-TP53-Clone3_Ctrl_input.bigWig<br>CPA-TP53-Clone3_Ctrl_H3K9me3_vs_CPA-TP53-Clone3_Ctrl_input.bigWig<br>CPA-TP53-Clone3_WGD_CTCF_vs_CPA-TP53-Clone3_WGD_input.bigWig<br>CPA-TP53-Clone3_WGD_H3K9me3_vs_CPA-TP53-Clone3_WGD_input.bigWig
Genome browser session<br>(e.g. UCSC)	No longer applicable.

Methodology

Replicates	One replicate for each condition.
Sequencing depth	Total paired-end reads after trimming, alignment, and PCR duplicates removal for samples generated for this study:<br><br>RPE_TP53_Ctrl_CTCF: 90,500,994<br>RPE_TP53_Ctrl_H3K27ac: 64,342,884<br>RPE_TP53_Ctrl_H3K27me3: 76,116,438<br>RPE_TP53_Ctrl_H3K4me3: 69,075,642<br>RPE_TP53_Ctrl_H3K9me3: 70,791,476<br><br>RPE_TP53_WGD_CTCF: 78,708,974<br>RPE_TP53_WGD_H3K9me3: 40,490,233<br><br>RPE_TP53_20w0T1_CTCF: 82,792,296<br>RPE_TP53_20w0T1_H3K27ac: 81,453,282<br>RPE_TP53_20w0T1_H3K27me3: 82,291,046<br>RPE_TP53_20w0T1_H3K4me3: 61,718,866<br>RPE_TP53_20w0T1_H3K9me3: 85,119,828<br><br>RPE_TP53_20w0T2_CTCF: 76,721,118

RPE_TP53_20w0T2_H3K27ac: 107,571,008
RPE_TP53_20w0T2_H3K27me3: 89,503,348
RPE_TP53_20w0T2_H3K4me3: 80,108,224
RPE_TP53_20w0T2_H3K9me3: 75,368,694

RPE_TP53_20w0T3_CTCF: 72,833,790
RPE_TP53_20w0T3_H3K27ac: 78,101,386
RPE_TP53_20w0T3_H3K27me3: 92,531,496
RPE_TP53_20w0T3_H3K4me3: 86,117,812
RPE_TP53_20w0T3_H3K9me3: 94,177,908

CPA-TP53-Clone3_Ctrl_CTCF: 29,735,520
CPA-TP53-Clone3_Ctrl_H3K9me3: 29,798,985
CPA-TP53-Clone3_WGD_CTCF: 29,683,395
CPA-TP53-Clone3_WGD_H3K9me3: 23,051,295

Antibodies

anti-Acetyl-Histone H3 (Lys27) (Cell Signaling Technology, catalogue no. 8173), anti-Tri-Methyl-Histone H3 (Lys9) (Cell Signaling Technology, catalogue no. 13969), anti-CTCF (Active Motif, catalogue no. 61311), anti-Tri-Methyl-Histone H3 (Lys4) (Cell Signaling Technology, catalogue no. 9751), Tri-Methyl-Histone H3 (Lys27) (Cell Signaling Technology, catalogue no. 9733). All antibodies were used at the concentrations recommended by the manufacturer.

Peak calling parameters

Peak calling for CTCF was done with MACS3 callpeak, version 3.0.0a6 with the following parameters:
--format BAMPE
--gsize hs
--nomodel
and the rest of the parameters left as default.

Data quality

- PCR duplicates were removed, FDR threshold of 0.05, minimum fold-change for a peak was set to 5

Software

- nfcore/chipseq pipeline 1.2.2
- deeptools, version 3.5.1
- MACS3, version 3.0.0a6

Flow Cytometry

Plots

Confirm that:

☒ The axis labels state the marker and fluorochrome used (e.g. CD4-FITC).

☒ The axis scales are clearly visible. Include numbers along axes only for bottom left plot of group (a 'group' is an analysis of identical markers).

☒ All plots are contour plots with outliers or pseudocolor plots.

☒ A numerical value for number of cells or percentage (with statistics) is provided.

Methodology

Sample preparation

Cells were collected and washed with PBS (Thermo Fisher Scientific, catalogue no. 10010023). Permeabilization was performed in 0.01% Triton-X100 (AppliChem, catalogue no. A1388) in PBS for 30-60 minutes at 4°C. Following PBS washes, the cells were fixed and stained with FxCycle™ PI/RNase Staining Solution (Thermo Fisher Scientific, catalogue no. F10797) overnight at 4°C in the absence of light.

Instrument

Guava® easyCyte™ (Luminex) and Galios (Beckman Coulter)

Software

FlowJo (BD).

Cell population abundance

All cells in the population were queried for PI-based DNA content.

Gating strategy

FSC/SSC used for exclusion of debris and FSC-H/FSC-W for the exclusion of doublets. No other gating strategy was necessary.

☒ Tick this box to confirm that a figure exemplifying the gating strategy is provided in the Supplementary Information.
